# London Prices of Drugs.—April 22

**Published:** 1814-05

**Authors:** 


### LONDON PRICES OF DRUGS.—April 22.

*(To be continued regularly.)*

| | £ | s. | d. | £ | s. | d. | per |
|---|---|---|---|---|---|---|---|
| Aloes Barbadoes, from | 20 | 10 | 0 to | 21 | 0 | 0 | C. |
| —— Cape | 10 | 0 | 0 .. | 11 | 0 | 0 | — |
| —— Succotrina | 20 | 0 | 0 .. | 0 | 0 | 0 | — |
| —— Epatica or E.I. | 14 | 0 | 0 .. | 16 | 0 | 0 | — |
| Angelica Root | 13 | 0 | 0 .. | 14 | 10 | 0 | — |
| Alkanet Root | 6 | 0 | 0 .. | 6 | 10 | 0 | — |
| Antimony Crude | 3 | 0 | 0 .. | 3 | 5 | 0 | — |
| Aqua Fortis | S. | 0 | 8 .. | D. | 1 | 1 | lb |
| Arrow Root fine | 0 | 2 | 0 .. | 0 | 3 | 6 | — |
| —— ordinary | 0 | 1 | 0 .. | 0 | 1 | 6 | — |
| Arsenic Red | 5 | 0 | 0 .. | 0 | 0 | 0 | C. |
| —— White | 3 | 3 | 0 .. | 0 | 0 | 0 | — |
| Balsam Capivi | 0 | 6 | 0 .. | 0 | 6 | 3 | lb. |
| —— Peru | 1 | 0 | 0 .. | 1 | 1 | 0 | — |
| —— Tolu | 0 | 14 | 0 .. | 0 | 14 | 6 | — |
| Bark Jesuits Com. | 0 | 3 | 6 .. | 0 | 4 | 0 | — |
| —— Second | 0 | 6 | 0 .. | 0 | 7 | 0 | — |
| —— Quil or best | 0 | 8 | 0 .. | 0 | 10 | 0 | — |
| —— Red | 0 | 7 | 0 .. | 0 | 10 | 0 | — |
| —— Yellow | 0 | 4 | 6 .. | 0 | 5 | 0 | — |
| Borax refined E.I. | none. | | | | | | |
| —— English | 0 | 3 | 9 .. | 0 | 4 | 0 | lb. |
| —— unrefined or Tinc. | 10 | 0 | 0 .. | 11 | 0 | 0 | C. |
| Camphire refined | 0 | 8 | 9 .. | 0 | 9 | 0 | lb. |
| —— unrefined | 28 | 0 | 0 .. | 32 | 0 | 0 | C. |
| Cantharides | 0 | 10 | 0 .. | 0 | 10 | 3 | lb. |
| Cardamoms (best) | 0 | 7 | 0 .. | 0 | 12 | 0 | — |
| Cassia Buds | 36 | 0 | 0 .. | } | 0 | 0 | C. |
| —— Fistula W.I. | 6 | 10 | 0 .. | in bond | | | — |
| —— Lignea | 25 | 0 | 0 .. | } | 0 | 0 | — |
| Castorum American | 2 | 10 | 0 .. | 2 | 15 | 0 | lb. |
| —— Russia | 11 | 0 | 0 .. | 0 | 0 | 0 | — |
| Castor Oil per bottle 1½ lb. | 0 | 3 | 9 .. | 0 | 4 | 3 | bo |
| Cocculus Indicus | 6 | 0 | 0 .. | 7 | 0 | 0 | C. |
| Colocynth Turkey | 0 | 5 | 0 .. | 0 | 5 | 6 | lb. |
| Columbo Root | 4 | 15 | 0 .. | 6 | 0 | 0 | C. |
| Cream of Tartar | 8 | 0 | 0 .. | 8 | 10 | 0 | — |
| Gallangal East-India | 5 | 0 | 0 .. | 0 | 0 | 0 | C. |
| Gentian Root | 3 | 5 | 0 .. | 3 | 6 | 0 | — |
| Ginseng | 0 | 1 | 6 .. | 0 | 0 | 0 | lb. |
| Grains of Guinea | 6 | 0 | 0 .. | 6 | 10 | 0 | C. |
| Gum Ammo. Drop | 30 | 0 | 0 .. | 0 | 0 | 0 | — |
| —— Lump | 8 | 0 | 0 .. | 9 | 0 | 0 | — |
| —— Animi | 3 | 10 | 0 .. | 7 | 10 | 0 | — |
| —— Arabic E.I. | 2 | 0 | 0 .. | 4 | 0 | 0 | — |
| —— Turkey Fine | 10 | 10 | 0 .. | 14 | 14 | 0 | — |
| —— Barbary | 7 | 7 | 0 .. | 8 | 0 | 0 | — |
| —— Assafœtida | 10 | 0 | 0 .. | 15 | 0 | 0 | — |
| —— Benjamin | 8 | 10 | 0 .. | 35 | 0 | 0 | — |
| —— Cambogium | 20 | 0 | 0 .. | 26 | 0 | 0 | — |
| —— Copal scraped | 0 | 3 | 0 .. | 0 | 4 | 6 | lb. |
| —— Galbanum | 16 | 0 | 0 .. | 20 | 0 | 0 | C. |
| Gum Guaiacum, from | 0 | 2 | 6 to | 0 | 3 | 0 | lb. |
| —— Mastic | 0 | 4 | 6 .. | 0 | 4 | 9 | — |
| —— Myrrh | 15 | 0 | 0 .. | 18 | 0 | 0 | C. |
| —— Olibanum | 6 | 0 | 0 .. | 7 | 0 | 0 | — |
| —— Oppoponax | 80 | 0 | 0 .. | 0 | 0 | 0 | — |
| —— Sandrac | 9 | 0 | 0 .. | 10 | 0 | 0 | — |
| —— Seneca Garbled | 5 | 2 | 0 .. | 5 | 5 | 0 | — |
| —— Tragacanth | 36 | 0 | 0 .. | 38 | 0 | 0 | — |
| Jalap | 0 | 5 | 0 .. | 0 | 5 | 3 | lb. |
| Ipecacuanha | 1 | 0 | 0 .. | 1 | 1 | 0 | — |
| Isinglass Book | 0 | 10 | 6 .. | 0 | 11 | 0 | — |
| —— Leaf | 0 | 10 | 6 .. | 0 | 11 | 0 | — |
| —— Long Staple | 0 | 10 | 6 .. | 0 | 11 | 0 | — |
| —— Short Staple | 0 | 10 | 6 .. | 0 | 11 | 0 | — |
| Manna Flakey | 0 | 5 | 3 .. | 0 | 5 | 6 | — |
| —— Sicily in Sorts | 0 | 4 | 0 .. | 0 | 4 | 3 | — |
| Musk China | 0 | 18 | 0 .. | 1 | 0 | 0 | oz. |
| Nux Vomica | 3 | 0 | 0 .. | 3 | 10 | 0 | C. |
| Oil of Vitriol | 0 | 0 | 3¾ | 0 | 0 | 0 | lb. |
| Opium East-India | 1 | 2 | 0 .. | 1 | 3 | 0 | — |
| —— Turkey | 1 | 9 | 0 .. | 1 | 10 | 0 | — |
| Pink Root | 0 | 4 | 0 .. | 0 | 4 | 6 | — |
| Quicksilver | 0 | 4 | 10 .. | 0 | 4 | 11 | — |
| Rhubarb East-India | 0 | 6 | 0 .. | 0 | 10 | 0 | — |
| —— Russia | 0 | 18 | 0 .. | 1 | 0 | 0 | — |
| Saffron Spanish | 2 | 5 | 0 .. | 2 | 10 | 0 | — |
| —— French | 1 | 15 | 0 .. | 2 | 0 | 0 | — |
| Sago | 7 | 0 | 0 .. | 8 | 0 | 0 | C. |
| Sal. Ammoniac | 10 | 10 | 0 .. | 11 | 0 | 0 | — |
| Sarsaparilla | 0 | 3 | 0 .. | 0 | 3 | 6 | lb |
| Sassafras | 7 | 0 | 0 .. | 8 | 0 | 0 | T. |
| Scammony Aleppo | 1 | 6 | 0 .. | 1 | 7 | 0 | lb. |
| —— Smyrna | 0 | 10 | 0 .. | 0 | 12 | 0 | — |
| Senna | 0 | 3 | 0 .. | 0 | 3 | 6 | — |
| Seeds Anni Alicant | 6 | 0 | 0 .. | 6 | 10 | 0 | C. |
| —— Coriander English | 1 | 10 | 0 .. | 1 | 16 | 0 | — |
| —— Cummin | 7 | 0 | 0 .. | 7 | 10 | 0 | — |
| —— Fenugreek | 1 | 10 | 0 .. | 1 | 13 | 0 | — |
| Shellack | 7 | 10 | 0 .. | 10 | 0 | 0 | — |
| Sticklack | 6 | 0 | 0 .. | 8 | 0 | 0 | — |
| Snake Root | 0 | 0 | 0 .. | 0 | 0 | 0 | lb. |
| Soap Castile or Spanish | 12 | 0 | 0 .. | 14 | 0 | 0 | C. |
| Spermaceti refined | 0 | 2 | 3 .. | 0 | 0 | 0 | lb. |
| Tamarinds West-India | 8 | 10 | 0 .. | 9 | 0 | 0 | C. |
| Tapioca Lisbon | 0 | 0 | 8 .. | 0 | 1 | 0 | lb. |
| Turmeric Bengal | 5 | 10 | 0 .. | 0 | 0 | 0 | C. |
| —— China | 6 | 10 | 0 .. | 7 | 0 | 0 | — |
| —— West-India | 8 | 0 | 0 .. | 9 | 0 | 0 | — |
| Verdigris Wet | 0 | 3 | 6 .. | 0 | 0 | 0 | lb. |
| —— Dry | | 5 | 0 .. | 0 | 5 | 2 | — |
| —— Crystallized | 0 | 8 | 6 .. | 0 | 9 | 0 | — |
| Vitriol Roman | 0 | 0 | 7 .. | 0 | 0 | 0 | — |
| —— Foreign white | 4 | 0 | 0 .. | 4 | 10 | 0 | C. |

### OBSERVATIONS.

At recent Sales of Merchandize by Auction, Messrs. Bowden and Tucker sold, on the 21st of April, 16 chests fine Pale Peruvian Bark, bonded, 7s. 5d. to 7s. 7d. per lb.—6 chests Rhubarb, 2s. 5d. to 5s. 7d. per lb.—4 bales Gum Tragacanth, 11l. per cwt.—20 bales Alkanet Root, 6l. 17s. 6d. per cwt.—3 chests and 2 casks Gum Arabic, 10l. to 11l. 15s. per cwt.—34 casks and 1 bag Lisbon Isinglass, 1s. 3d. to 2s. 2d. per lb.—1 chest Liquorice, 10l. per cwt.—1 chest Gum Galbanum, 10l. 10s. per cwt—10 stands Burgundy Pitch, 75s. per cwt.

